# HPV Virus Transcriptional Status Assessment in a Case of Sinonasal Carcinoma

**DOI:** 10.3390/ijms19030883

**Published:** 2018-03-16

**Authors:** Gennaro Ilardi, Daniela Russo, Silvia Varricchio, Giovanni Salzano, Giovanni Dell’Aversana Orabona, Virginia Napolitano, Rosa Maria Di Crescenzo, Alessandra Borzillo, Francesco Martino, Francesco Merolla, Massimo Mascolo, Stefania Staibano

**Affiliations:** 1 Department of Advanced Biomedical Sciences, Pathology Section, University of Naples “Federico II”, 80131 Naples, Italy; gennaro.ilardi@unina.it (G.I.); danielarusso83@yahoo.it (D.R.); silvia.varricchio@gmail.com (S.V.); virnap87@hotmail.it (V.N.); rosamariadicrescenzo@gmail.com (R.M.D.C.); alessandretta82@yahoo.it (A.B.); f_martino@live.it (F.M.); mmascol@gmail.com (M.M.); staibano@unina.it (S.S.); 2 Department of Neuroscience and Reproductive and Odontostomatological Sciences, Operative Unit of Maxillo-Facial Surgery, University of Naples “Federico II”, 80131 Naples, Italy; giovannisalzanomd@gmail.com (G.S.); giovanni.dellaversanaorabona@unina.it (G.D.O.); 3 Department of Medicine and Health Sciences “V. Tiberio”, University of Molise, 86100 Campobasso, Italy

**Keywords:** HPV, sinonasal carcinoma, RNAscope^®^, INNO-Lipa, p16INK4a

## Abstract

Human Papilloma Virus (HPV) can play a causative role in the development of sinonasal tract malignancies. In fact, HPV may be the most significant causative agent implicated in sinonasal tumorigenesis and is implicated in as many as 21% of sinonasal carcinomas. To date, there are no definitive, reliable and cost-effective, diagnostic tests approved by the FDA for the unequivocal determination of HPV status in head and neck cancers. We followed an exhaustive algorithm to correctly test HPV infection, including a sequential approach with p16INK4a IHC, viral DNA genotyping and in situ hybridization for E6/E7 mRNA. Here, we report a case of sinonasal carcinoma with discordant results using HPV test assays. The tumor we describe showed an irregular immunoreactivity for p16INK4a, and it tested positive for HPV DNA; nevertheless, it was negative for HR-HPV mRNA. We discuss the possible meaning of this discrepancy. It would be advisable to test HPV transcriptional status of sinonasal carcinoma on a diagnostic routine basis, not only by p16INK4a IHC assay, but also by HPV DNA genotyping and HR-HPV mRNA assessment.

## 1. Introduction

Squamous cell carcinoma (SCC) is the most common form of malignant neoplasia in the sinonasal tract, the greatest risk factor in some being smoking tobacco, followed by exposure to solvents, wood dusts, leather dusts and high-risk Human Papilloma Virus (HPV) infection.

In 2017 the WHO (World Health Organization) updated the classification of Head and Neck Tumors, also revising the chapter on tumors of the nasal cavity, paranasal sinuses and skull base, introducing new entities such as HPV-related sinonasal carcinomas [1]. 

Sinonasal squamous cell carcinomas are further sub-classified into keratinizing and non-keratinizing squamous cell carcinomas (KSCC versus NKSCC).

Sinonasal KSCC and NKSCC show significant differences in their clinical, pathological and molecular characteristics, and therefore, their recognition is relevant [1]. Transcriptionally active high-risk HPV is more frequently detected in NKSCC than in KSCC (35–50% and 0–15%, respectively) [2,3,4], although the clinical significance of HPV positivity in the sinonasal SCC has not been conclusively assessed as it has been for the oropharynx [5,6]. The expression of HPV high-risk early genes, E1 and E2, can repress HPV high-risk-dependent transformation [7]. It has been shown that expression of E2 from intact episomes can regulate the expression of E6/E7 gene products from the integrated viral genomes via an *in trans* mechanism [8,9]. The E2 gene disruption, usually occurring in case of viral integration in the host cell genome, results in overexpression of E6/E7 gene products [10,11].

Several authors have recently pointed out that the sinonasal tract is the second anatomical site for HR-HPV related tumors to arise, more than a fifth of which show a transcriptionally active HPV [12,13,14,15].

Some reports seem to support the hypothesis of a better outcome for HPV-related sinonasal carcinomas [5,16,17]

Nevertheless, there is an urgent need to collect more data in order to sort out the role of HR-HPV infection on the biological behavior of sinonasal SCC. 

Several HPV assays are currently available, but none offer optimal sensitivity and specificity levels for HPV detection when used as a single test [18]. In order to correctly test the HPV infection status in solid tumors other than SCC of the uterine cervix, it would be advisable to follow a more exhaustive algorithm including a sequential approach with p16INK4a IHC, viral DNA genotyping and insitu hybridization for E6/E7 mRNA [19].

Here, we report a case of sinonasal carcinoma with discordant results at HPV test assays. The tumor we describe showed an irregular immunoreactivity for p16INK4a, and it resulted positive for HPV DNA; nevertheless, it was negative for HR-HPV mRNA. We discuss the possible meaning of this discrepancy.

## 2. Case Presentation

A 63-year-old male patient with a past medical history of a complete and permanent obstruction of the left nasal cavity for the past 1 year, during which he received a contrast-enhanced free computed tomography (CT) scan, which was not diagnostic, and had not received any effective medical treatments, presented to the emergency room for a sudden difficulty in breathing. The patient was sent to the Maxillofacial Surgery Department, where he revealed that he had also performed a previous biopsy of the nasal mass that received a histological diagnosis of sinonasal papilloma with suspected virus-related cytopathic changes. On examination, edema of the left periorbital region was detected, with exophthalmos of the left eye with limitation of visual field in the upper and outer quadrants. The anterior rhinoscopy revealed a blushed mucosa, no flow through the left nasal cavity, and convex right deviation of the nasal septum. The patient did not refer hyposmia. Due to these findings, the patient underwent a contrast-enhanced magnetic resonance imaging (MRI) scan. The MRI scan confirmed the presence of a lesion occupying the left nasal cavity, associated with a displacing erosion of the surrounding bone walls, extending laterally into the left maxillary sinus, superiorly in the context of the ethmoid cells, posteriorly reaching the sphenoid sinus, supero-laterally protruding inside the left orbitary cavity, infiltrating the lateral and inferior rectus muscles and displacing the optic nerve upwards. The lesion was irregularly enhanced upon contrast medium administration. These findings suggested a diagnosis of a malignant neoplasia. 

The lesion was biopsied, and the pathology report highlighted the presence of a carcinoma in situ.

The patient underwent surgery for resection with left hemimaxillectomy, turbinectomy, exenteratio orbitae and subsequent reconstruction with temporal muscle flap and apposition of orbital filling implants. 

**Figure 1:**
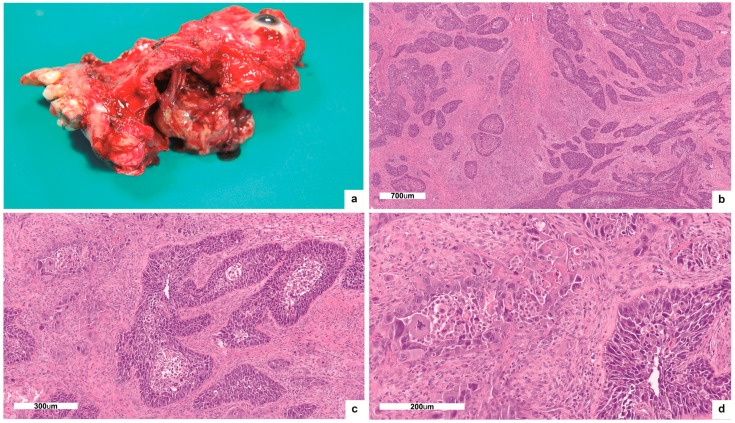
Gross examination and H and E stain: (**a**) Gross examination of left hemimaxillectomy with turbinectomy and exenteratio orbitae revealed a nodular lesion macroscopically occupying the maxillary sinus and infiltrating the orbital floor; (**b**,**c**,**d**) H-and-E-stained tissue sections demonstrated a poorly differentiated, infiltrating carcinoma (b:50×; c:100×; d:200×).

At gross examination of the specimen, we observed a nodular, whitish lesion of hard-elastic consistency, measuring 4 × 3.5 cm on the two-major axis, macroscopically occupying the maxillary sinus, and infiltrating the orbital floor (Figure 1a). Close to the lesion, a polypoid formation of 4 × 4 cm of friable appearance was detected. A month after surgery, the patient died due to post-operative complications.

## 3. Results

**Figure 2:**
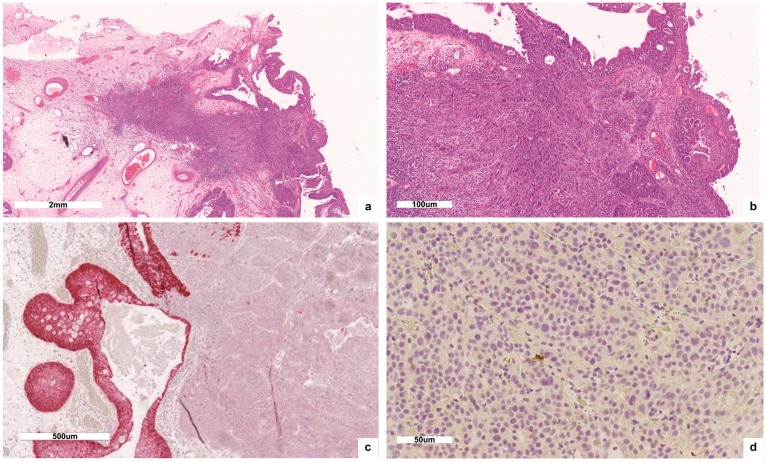
(**a**,**b**) The second described polypoid lesion showed multifocal areas of carcinoma in situ, only focally invasive (a:50×; b:100×); (**c**) IHC signal of p16INK4a (100×); (**d**) mRNA ISH assay negative for E6/E7 mRNA (200×).

H-and-E-stained tissue sections demonstrated a poorly differentiated, infiltrating carcinoma, involving the maxillary bone and the orbital floor, reaching the border of the orbital striated muscles (Figure 1b–d). The optic nerve and ocular tissues were free of neoplastic invasion. The second polypoid lesion showed multifocal areas of carcinoma in situ, only focally invasive (Figure 2a,b). IHC showed irregular, “patched” positivity for p16INK4a, as immunostaining was not always uniformly distributed across the tumor mass. In particular, the IHC signal of p16INK4a was convincingly positive with nuclear and cytoplasmic staining only in the areas of carcinoma in situ, but not in the infiltrating ones (Figure 2c). The lesion proved to be positive for HPV 16 DNA presence at the genotyping of viral DNA with INNO-LiPA assay. On the contrary, mRNA ISH assay did not identify E6/E7 mRNA (Figure 2d). So, we concluded that the tumor harbored a non-transcriptional active HPV infection.

## 4. Discussion

To date, the FDA has not yet approved a reliable diagnostic test to assess the HPV status in head and neck cancers. Different techniques are currently used for HPV detection in solid tumors, ranging from specific PCR methods, real-time PCR tests, in situ DNA hybridization (ISH) and immunohistochemical detection of surrogate biomarkers (protein p16INK4a). Since none of these methods offers optimal levels of sensitivity and specificity, there is a need for a multitest approach that can help to unravel discordant results [20]. 

HPV also plays a causative role in the development of sinonasal tract malignancies, accounting for as many as 21% of sinonasal carcinomas [5].

A crucial role has been recently ascribed to the distinction between HPV-active and HPV-inactive related tumors in those anatomical sites classically associated with HPV infection, such as oropharynx and uterine cervix. Persistent HPV infection is recognized as a causative event in oropharyngeal squamous cell carcinomas [21], and tumor HPV status is a strong and independent prognostic factor for survival of oropharyngeal Squamous Cell Carcinoma (OPSCC) patients [22], while the role of HPV infection in non-oropharyngeal SCC is still debated [23,24]. To date, beyond HPV, there are no reliable prognostic or predictive biomarkers, especially for non-oropharyngeal SCC [25,26]. HPV-active tumors are those positive for p16 immunohistochemistry and for HPV RNA and DNA, while HPV-inactive ones are positive for HPV DNA and negative for HPV RNA [27]. The gene expression profiles are also different in the two groups. HPV-negative tumors are characterized by the expression of genes that control cell motility, angiogenesis and epithelial–mesenchymal transition; all of which determine aggressive clinical behavior [28]. HPV-active tumors have gene expression profiles that clearly show a “HPV signature,” dominated by changes in genes that control cell cycle, proliferation, and mitosis [28]. The gene expression profiles of inactive HPV tumors lack the "HPV signature" features and are, therefore, more similar to HPV-negative tumors, although some differences can be detected, the clinical significance of which remains to be determined [28,29]. Currently, HPV DNA in inactive HPV tumors is interpreted as a simple passenger not involved in tumor progression. Tomar et al. have postulated that HPV-inactive tumors may originate from HPV-active lesions, which progressively become independent of the action of E6/E7 oncoproteins for neoplastic proliferation and progression [29]. Silencing of E6/E7 genes can occur due to methylation phenomena in cells that have acquired mutations that can “replace” their function. However, the mechanisms underlying the acquisition of HPV-inactive tumor virus independence are still under investigation [29]. Transient expression of HPV oncogenic proteins has been reported for genus beta HPV in non-melanoma skin cancer [30]. Genus beta HPV is not required for the maintenance of a tumor, although it is involved in the first step of neoplastic transformation, probably due to its involvement in DNA damage repair, destabilizing keratinocytes upon UV exposure [31]

Here, we report a case of head and neck carcinoma with discordant results that emerged from HPV tests. It showed irregular positivity for p16INK4a, positivity for HPV 16 DNA, but negativity for HR-HPV mRNA. Although the data in the literature concerning HPV in the sinonasal tract are not univocal, we believe that even in our unusual case we can hypothesize an initial HPV infection, as evidenced by the previous diagnosis of papilloma, and the positivity for protein p16INK4a of carcinoma in situ. Probably, with the advancement of the neoplasia, the latter became independent of the action of the virus, acquiring a more aggressive phenotype [32].

This case report underlines the importance, in case of suspected HPV infection, to identify the viral transcription status. A key point for this alternative approach is based on the recently proposed modification of RNA in situ hybridization (RNAISH) assay designed to test the transcriptional activity of HPV, detecting E6/E7 mRNA, within tumor cells, of the most common high-risk HPV types via a colorimetric method (RNAscope^®^) [19,33]. This assay is easy to interpret, readily adaptable to the clinical laboratory, and provides direct evidence of HPV transcriptional activity on a routine formalin-fixed paraffin-embedded tissue section with the great advantage to overlay the resulting signal with tissue morphology. This test demonstrates transcriptionally active oncogenic HPVs with an excellent sensitivity and specificity against the reference standard [3]; RNAscope^®^ could stably complement the current diagnostic algorithms for HPV-related cancer, precisely identifying HPV-driven tumors and efficiently distinguishing between HPV-active and HPV-inactive ones. 

The multi test protocol we applied proved to clearly distinguish, on the basis of HPV status, the infiltrating lesion from the papillary, mostly non-infiltrating one. Interestingly, in the latter, the CIS component showed a strong p16INK4a positivity, and lacked it in the infiltrating fraction. We hypothesize an original causative role for high-risk HPV in the onset of the neoplasia, and a progressive lack of dependencies from HPV-related proliferative stimuli during neoplastic progression towards a more aggressive, infiltrating phenotype. There is an urgent need for more data about the clinical outcome and biological behavior of HPV-related carcinomas, in order to unravel the role of viral infection as a prognostic biomarker and predictive of chemo and radiotherapy responses. To date, it is believed that estimates about sinonasal carcinomas HPV status could be inaccurate, since guidelines do not recommend a routine assessment of HPV status in this region and, most importantly, the methods of detection are still extremely variable among different laboratories, with most of the tests used for screening purposes not assessing the transcriptional activity of the virus.

## 5. Materials and Methods

Formalin fixed, paraffin-embedded specimens were processed for routine H and E staining. Three different tests were performed for HPV detection: p16 immunohistochemistry (IHC) staining, INNO-LiPA HPV Genotyping v2 Extra test to genotype HPV DNA, and HR-HPV E6/E7 mRNA ISH assay (RNAscope^®^, Advanced Cell Diagnostics, Milano, Italy) to confirm the presence of HPV in tumor cells and its transcriptional activity. 

All subjects gave their informed consent for inclusion before they participated in the study. The study was conducted in accordance with the Declaration of Helsinki, and the protocol was approved by the AOU “Federico II” of Naples (Approval date: 27 February 2013). 

### 5.1. p16INK4a Immunohistochemistry

p16INK4a-IHC was performed on a Ventana Benchmark Ultra (Ventana Medical Systems Inc., Tucson, AZ, USA) using the CINtec p16 Histology Kit (Ref. 9511, Roche mtm laboratories AG, Heidelberg, Germany), according to both manufacturers’ recommendations. The previously prepared 4 μm tumor FFPE tissues paraffin sections were deparaffinized and subjected to antigen retrieval using CC1 buffer for 30 min. Subsequently they were consecutively incubated in the prediluted CINtec p16 primary antibody (clone E6H4) for 20 min at room temperature and revealed with Ultra View Universal Alkaline Phosphatase Red Detection Kit (Ventana Medical Systems, Inc, Tucson, AZ, USA).Slides were finally counterstained with Hematoxylin II for 8 min (Ventana Medical Systems, Inc., Tucson, AZ, USA) and Bluing reagent for 4 min and then washed. p16INK4a IHC was scored as positive if there was strong, homogeneous and diffuse nuclear and cytoplasmic staining present in greater than 70% of the malignant cells [34]. 

### 5.2. HPV Genotyping

For INNO-LiPA HPV Genotyping v2 Extra test, DNA was extracted with QIAmp DNA Mini Kit (Qiagen, Hilden, Germany) following the manufacturer’s instructions and eluted in 100 μL as suggested in the INNO-LiPA assay manufacturer’s instructions. Genomic DNA was analyzed for the presence of specific HPV types by multiplex PCR-based assay (SPF10-modified primers), followed by reverse line blot hybridization, detects and genotypes 28 different HPV genotypes, including 18 high-risk HPVs (16, 18, 26, 31, 33, 35, 39, 45, 51, 52, 53, 56, 58, 59, 66, 68, 73, 82), 6 low-risk HPVs (6, 11, 40, 43, 44, 54, 70), and 3 other not-classified HPVs (69, 71, 74), targeting a 65-bp fragment of the L1 ORF. The assay was carried out following the manufacturer’s instructions. The results were visually interpreted by two independent investigators by comparing them with a template provided with the assay [35]. 

### 5.3. RNAscope^®^

**Figure 3:**
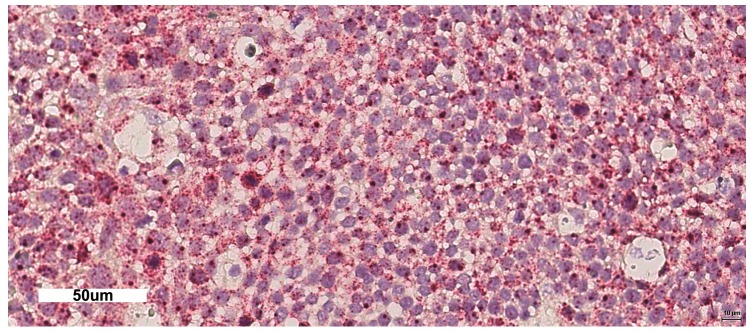
mRNA ISH assay positive control. PPIB mRNA was tested in the sample shown in Figure 2b (200×).

HR-HPV E6/E7 mRNA ISH assay was performed using the automated RNAscope^®^ 2.5 LS RED assay kit (Cat# 322150 Advanced Cell Diagnostics, S.r.l., Segrate (Milano), Italy) on the Leica Biosystems BOND RX platform (LS) and the HPV-HR16 Probe (Cat# 311521, Advanced Cell Diagnostics) in accordance with the manufacturer’s instructions. Briefly, 4 μm sections were baked and deparaffinized on the instrument, followed by target retrieval (15 min at 95 °C using Leica Epitope Retrieval Buffer 2) and protease treatment (15 min at 40 °C). Probes were then hybridized for 2 h at 42 °C followed by RNAscope amplification. Signal detection was performed using fast red (red). Peptidylprolyl isomerase B (PPIB, a constitutively expressed endogenous gene) (Figure 3) and the bacterial gene, dapB, were used as positive and negative controls, respectively. The PPIB test was used to assess the presence of hybridizable RNA to confirm adequate RNA quality and was defined as adequate if there were at least 5 punctate signal dots in most tumor cells in the section. This is especially important when the HPV probe signal is negative to avoid a false-negative result. The dapB test was used to assess nonspecific staining; only those cases that were negative or weakly stained were considered for HPV scoring. A positive HPV test result was defined as punctate red signals that localized to the cytoplasm and/or nucleus of any of the malignant cells, and where staining was present in the negative control, it was at least three times as strong as the dapB staining.

## 6. Conclusions

It would be advisable, in our opinion, to test the HPV status of sinonasal carcinoma on a diagnostic routine basis, not only by p16INK4a IHC assay, but taking advantage of those techniques allowing a reliable determination of viral transcriptional status. The RNAscope^®^ technique allows to test the HPV transcriptional status via the assessment of E6/E7 mRNAs, the only two HPV viral genes with a recognized oncogenic potential at the cellular level and preserving the tumor morphology on the glass slide.

## Abbreviations

HPV	Human Papilloma Virus
OSCC	Oral Squamous Cell Carcinoma
OPSCC	Oropharyngeal Squamous Cell Carcinoma
FFPE	Formalin Fixed Paraffin Embedded
ISH	In Situ Hybridization
FDA	Food and Drug Administration
ICH	Immunohistochemistry
HR	High Risk
UV	Ultraviolet
